# Supplementary material for: Many-body enhancement of high-harmonic generation in monolayer MoS2
Source: Nat Commun. 2024 Jul 24;15:6228. doi: 10.1038/s41467-024-50534-3 (PMC11266681; doi:10.1038/s41467-024-50534-3)
Supplement: Supplementary file 1 — Supplementary Information [file 41467_2024_50534_MOESM1_ESM.pdf]

Many-Body Enhancement of High-harmonic Generation in Monolayer MoS_2

Supplementary information

Victor Chang Lee,[1,2] Lun Yue,[3] Mette B. Gaarde,[3]
Yang-hao Chan,[4,5,*] and Diana Y. Qiu[1,2,†]

[1]*Department of Mechanical Engineering and Materials Science, Yale University, New Haven, CT 06520, USA*

[2]*Energy Science Institute, Yale University, New Haven, CT 06520, USA*

[3]*Department of Physics and Astronomy, Louisiana State University, Baton Rouge, LA 70803, USA*

[4]*Institute of Atomic and Molecular Sciences, Academia Sinica, Taipei 10617, Taiwan*

[5]*Physics Division, National Center of Theoretical Sciences, Taipei 10617, Taiwan*

(Dated: July 22, 2024)

* yanghao@gate.sinica.edu.tw

† diana.qiu@yale.edu

SUPPLEMENTARY NOTE 1: STATIC-COHSEX APPROXIMATION

In the time-dependent adiabatic GW approximation (TD-aGW), we assume that the dynamical effects in extended systems are very small and therefore[1], the self-energy operator in the Keldysh Green's function formalism can be decoupled and split into the dynamical effects at equilibrium Σ^{GW} and an instantaneous correction $\delta\Sigma(t)$ which is calculated within the Coulomb-hole static screened-exchange (static-COHSEX) approximation. The equilibrium self-energy is well-approximated by the static GW self-energy $\Sigma = iGW$, where G is the electronic Green's function constructed from the DFT orbital in a one-shot non-self consistent approach and W is the Coulomb interaction in the random phase approximation. This approximation is also known as G_0W_0.

The instantaneous correction $\delta\Sigma(t)$ is calculated using the static-COHSEX approximation[2–4] which consist in separating the self-energy into two parts $\Sigma(t) = \Sigma^{SEX}(t)+\Sigma^{COH}(t)$ where

$$\Sigma^{SEX}_{nm,\mathbf{k}}(t) = i\sum_{n'm'} G_{n'm',\mathbf{k}'}(t)W_{nn'mm',\mathbf{k},\mathbf{k}'} \tag{1}$$

is the screened-exchange operator and

$$\Sigma^{COH}_{nm,\mathbf{k}}(t) = -\sum_{n'm'} W_{nn'mm',\mathbf{k},\mathbf{k}'}\frac{1}{2}\delta(\mathbf{k}-\mathbf{k}') \tag{2}$$

is the Coulomb-hole operator. In this approximation, W is the screened Coulomb interaction at equilibrium computed using the random phase approximation and therefore it does not depend on $G_{n'm',\mathbf{k}'}(t)$. On the other hand, the screened-exchange is recalculated at each time-step. For the case of HHG, we expect that this approximation is reasonable since the excited state population is very small and should have a negligible contribution to the screening.

SUPPLEMENTARY NOTE 2: COMPUTATIONAL DETAILS

Mean-field density functional theory (DFT) calculations within the semilocal generalized gradient approximation of Perdew, Burke, and Ernzerhoff (PBE) were performed using the Quantum Espresso code.[5] The calculations including spin-orbit interactions were done in the primitive cell of monolayer MoS_2. Core electrons were replaced by a norm-conserving pseudopotential obtained from the ONCV SG15 library.[6] A planewave cutoff energy of 80 Ry and a 12×12×1 k-grid was used in the self-consistent field (scf) calculations. The distance between the repeated cells in the out-of-plane direction was 15Å. The fully relax lattice parameter of the unit cell was found to be $3.168\mathring{A}$, which is in good agreement with previous theoretical calculations and deviates less than 1% from the experimental lattice constant.[7–10]

One-shot GW calculations using the BerkeleyGW package were performed on top of the DFT calculations to obtain the equilibrium quasiparticle energies.[11] We employed 6000 unoccupied states, dielectric cutoff of 45 Ry and a $\mathbf{k}$ point grid of $6 \times 6 \times 1$ with a nonuniform subsampling scheme of 10 $\mathbf{q}$ points, resulting in an effective k-point sampling of greater than $60 \times 60 \times 1$, to converge the quasiparticle energies.[12] Spin-orbit coupling was included within a fully-relativistic spinor formalism [13], and the frequency-dependence of the self-energy is included within the Hybertsen-Louie generalized plasmon pole model (HL-GPP) [2]. The GW plus and Bethe Salpeter equation (GW-BSE) [14] approach within the Tamm-Dancoff approximation, including 8 valence bands and 6 conduction bands on a $36 \times 36 \times 1$ $\mathbf{k}$ grid, was used to obtain the linear optical absorption spectra and the electron-hole interaction matrix elements used as input for the TD-aGW approach.

Below we discuss the convergence of the number of bands and the $\mathbf{k}$ grid at the IP@DFT level.

While the main physics in the high-harmonic generation (HHG) spectra are captured with a 2 valence band 2 conduction band (considering spin-orbit coupling), Lun *et al.* showed that the higher energy harmonics required the inclusion of higher energy conduction bands to be correctly described.[15] In Supplementary Fig. 1, we show the convergence of the HHG spectra at the IP@DFT level as a function of the number of conduction bands and as function of the number of valence bands. Our convergence results shows that 6 conduction bands are required to converge the spectra at higher energies and increasing the number of

valence bands to 8 has a small effect on the harmonic peak at 3.35 eV. Therefore, we used 8 valence bands and 6 conduction bands in our TD-aGW calculations.

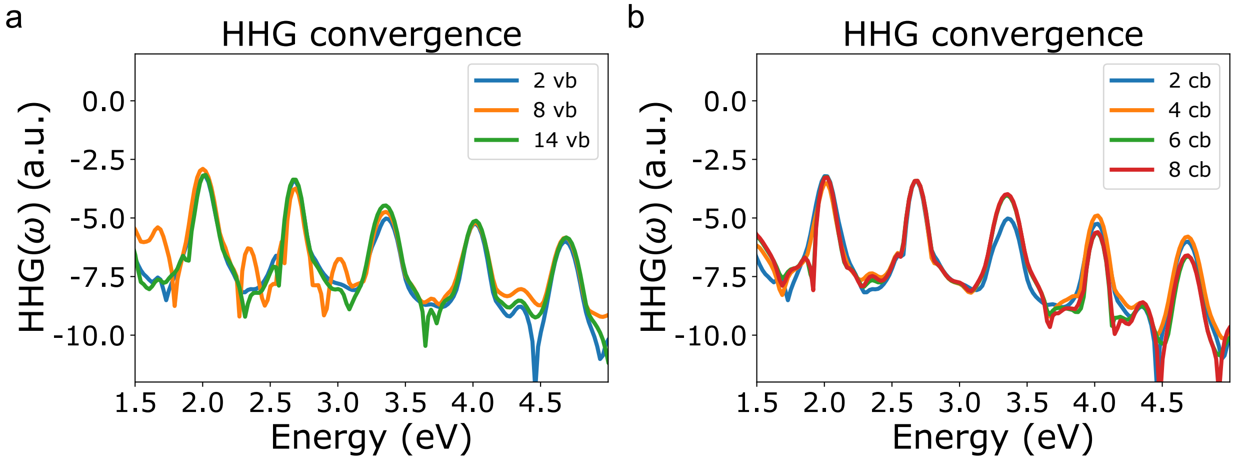

Supplementary Fig. 1. Convergence of **a**) the number of valence bands (vb) and **b**) conduction bands (cb) of the high harmonic generation spectra at the IP@DFT level. The HHG intensity is presented in logarithmic scale and in atomic units (a.u.)

In Supplementary Fig. 2 we show the convergence of the **k** grid. While the higher harmonics required a denser **k** grid sampling, the main HHG spectra is well described by the less dense 36×36×1 **k** grid. Therefore, we performed the TD-aGW calculations on a 36×36×1 Γ-centered **k** grid. We note that finite k-point sampling may slightly break the symmetry when the driving field is along a direction incommensurate with the uniform k-point grid.

The external driving field was given by

$$\mathbf{E}(t) = A \sin\left(\frac{\pi t}{T}\right)^2 \sin(\omega t)\, \mathbf{P}, \tag{3}$$

where A corresponds to the intensity of a light, T is the duration of the light pulse, ω is the frequency of the light and $\mathbf{P}$ is the polarization of the light. We use the Wannier90 code to calculate the overlaps required for rotation of the wavefunctions to a locally smooth gauge.[16] Time-integration is performed with a time step of 0.05 fs using a fourth order Runge-Kutta method. Relaxation processes are described by a diagonal term measured to be approximately approximately 25 meV.[17] Dephasing times in this work is set to 13.8 fs (300 meV) which is in the range of previous works from very short times of 1 fs (4 eV) to 78 fs (53 meV) [18–21]

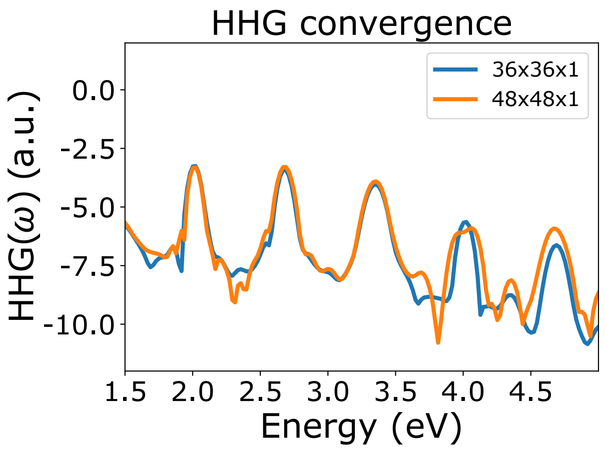

Supplementary Fig. 2. Convergence of **k** grid sampling for the high harmonic generation spectra at the IP@DFT level. The HHG intensity is presented in logarithmic scale and in atomic units (a.u.)

SUPPLEMENTARY NOTE 3: BAND PROJECTED BERRY CURVATURE

The **k** space Berry curvature of the top valence band and bottom conduction band shown in Fig. 5 in the main manuscript is calculated using the Wannier90 code and the Wannier-Berri post processing tool.[16, 22] Here, in Supplementary Fig. 3, we show the projected Berry curvature onto the $K' - \Gamma - K$ and $K' - M - K$ paths.

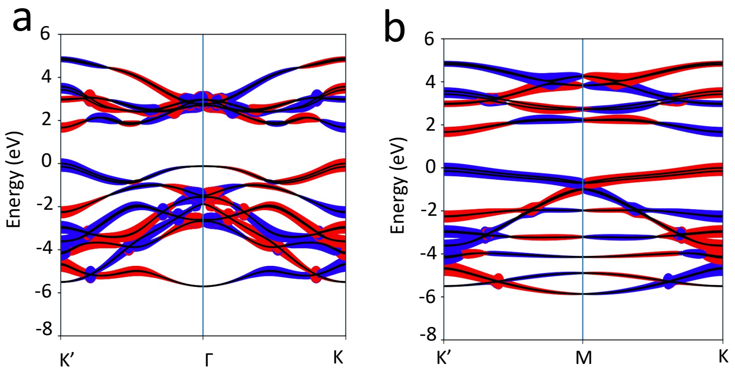

Supplementary Fig. 3. Berry curvature of monolayer MoS2 projected onto the DFT band structure along **a**) the $K' - \Gamma - K$ path and **b**) the $K' - M - K$ path. Positive(negative) values are shown in red(blue).

SUPPLEMENTARY NOTE 4: ELECTRON-HOLE CREATION AND RECOMBINATION

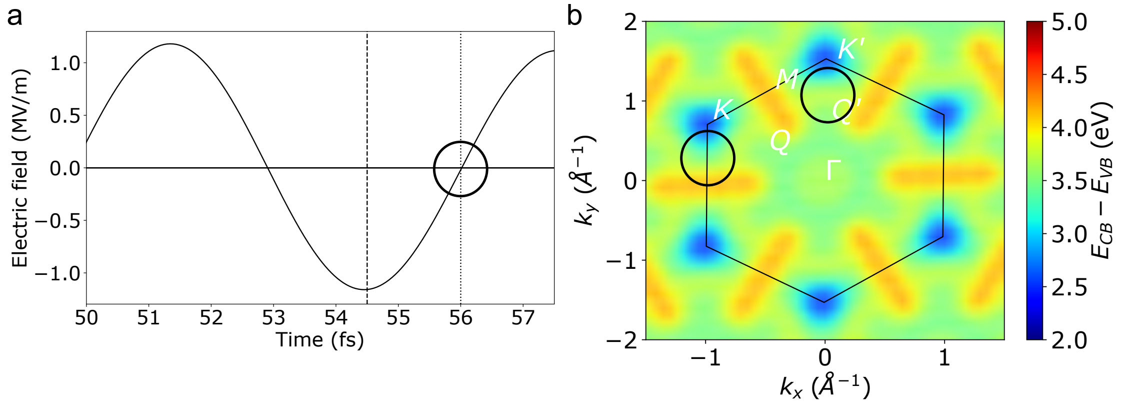

Supplementary Fig. 4. **a**)Electric field of the light as function of time for a laser pulse as described in the main text . Vertical dashed and dotted line indicate the times shown in Fig.5 in the main manuscript. **b**) Difference between the conduction band E_{CB} and valence band E_{VB} quasiparticle energies computed using the GW approximation. Black circles denote the electron and hole recombination peak.

SUPPLEMENTARY NOTE 5: ELECTRONIC POPULATION FROM IP MODELS

Supplementary Fig. 5 contains the electronic occupations in **k**-space from the high harmonic simulation at an angle of 30° and a photon energy of 0.335 eV simulated using the IP@GW and IP@DFT approximations. The difference in electronic occupation between the TD-aGW model and the IP@GW or IP@DFT is also presented.

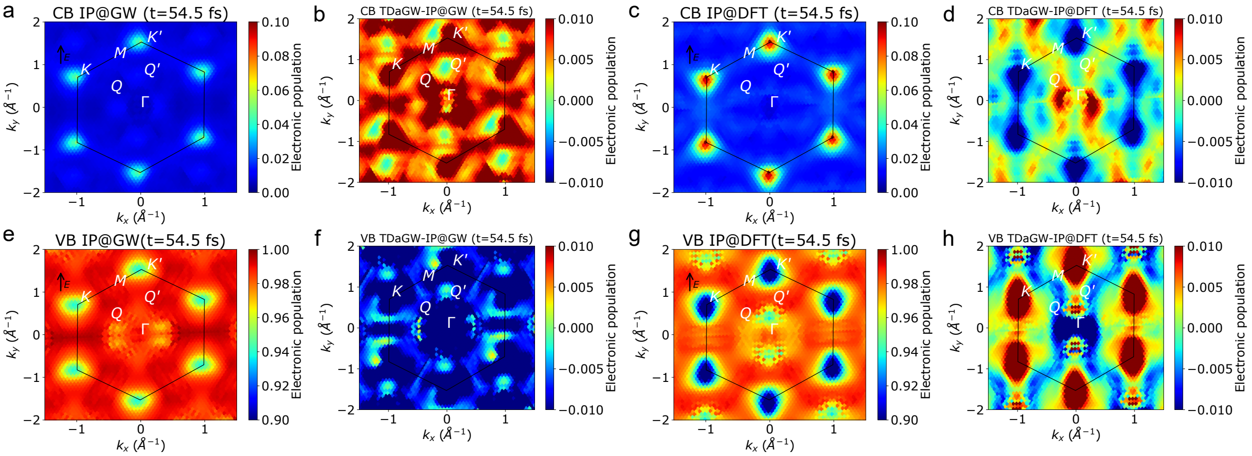

Supplementary Fig. 5. Electron population in **k** space of the bottom **a**) conduction band and **e**) top valence band computed using IP@GW. **b**) and **f**) shows the difference in electronic population between the TD-aGW and IP@GW approximations. **c-d**) and **g-h**) the same using IP@DFT.

[1] Andrea Marini and Rodolfo Del Sole, "Dynamical excitonic effects in metals and semiconductors," Phys. Rev. Lett. **91**, 176402 (2003).

[2] Mark S. Hybertsen and Steven G. Louie, "Electron correlation in semiconductors and insulators: Band gaps and quasiparticle energies," Phys. Rev. B **34**, 5390–5413 (1986).

[3] C. Attaccalite, M. Gruning, and A. Marini, "Real-time approach to the optical properties of solids and nanostructures: Time-dependent bethe-salpeter equation," Phys. Rev. B **84**, 245110 (2011).

[4] Jack Deslippe, Georgy Samsonidze, Manish Jain, Marvin L. Cohen, and Steven G. Louie, "Coulomb-hole summations and energies for *gw* calculations with limited number of empty orbitals: A modified static remainder approach," Phys. Rev. B **87**, 165124 (2013).

[5] Paolo Giannozzi, Stefano Baroni, Nicola Bonini, Matteo Calandra, Roberto Car, Carlo Cavazzoni, Davide Ceresoli, Guido L Chiarotti, Matteo Cococcioni, Ismaila Dabo, Andrea Dal Corso, Stefano de Gironcoli, Stefano Fabris, Guido Fratesi, Ralph Gebauer, Uwe Gerstmann, Christos Gougoussis, Anton Kokalj, Michele Lazzeri, Layla Martin-Samos, Nicola Marzari, Francesco Mauri, Riccardo Mazzarello, Stefano Paolini, Alfredo Pasquarello, Lorenzo Paulatto, Carlo Sbraccia, Sandro Scandolo, Gabriele Sclauzero, Ari P Seitsonen, Alexander Smogunov, Paolo Umari, and Renata M Wentzcovitch, "Quantum espresso: a modular and open-source software project for quantum simulations of materials," Journal of Physics: Condensed Matter **21**, 395502 (2009).

[6] Peter Scherpelz, Marco Govoni, Ikutaro Hamada, and Giulia Galli, "Implementation and validation of fully relativistic gw calculations: Spin–orbit coupling in molecules, nanocrystals, and solids," Journal of Chemical Theory and Computation **12**, 3523–3544 (2016).

[7] Hai He, Pengfei Lu, Liyuan Wu, Chunfang Zhang, Yuxin Song, Pengfei Guan, and Shumin Wang, "Structural properties and phase transition of na adsorption on monolayer mos2," Nanoscale Research Letters **11**, 330 (2016).

[8] Francis Opoku, Krishna Kuben Govender, Cornelia Gertina Catharina Elizabeth van Sittert, and Penny Poomani Govender, "Role of mos2 and ws2 monolayers on photocatalytic hydrogen production and the pollutant degradation of monoclinic bivo4: a first-principles study," New J. Chem. **41**, 11701–11713 (2017).

[9] P A Young, "Lattice parameter measurements on molybdenum disulphide," Journal of Physics D: Applied Physics **1**, 936 (1968).

[10] Yu Xiang, Xin Sun, Lukas Valdman, Fu Zhang, Tanushree H Choudhury, Mikhail Chubarov, Joshua A Robinson, Joan M Redwing, Mauricio Terrones, Yuan Ma, Lei Gao, Morris A Washington, Toh-Ming Lu, and Gwo-Ching Wang, "Monolayer mos2 on sapphire: an azimuthal reflection high-energy electron diffraction perspective," 2D Materials **8**, 025003 (2020).

[11] Jack Deslippe, Georgy Samsonidze, David A. Strubbe, Manish Jain, Marvin L. Cohen, and Steven G. Louie, "Berkeleygw: A massively parallel computer package for the calculation of the quasiparticle and optical properties of materials and nanostructures," Computer Physics Communications **183**, 1269–1289 (2012).

[12] Felipe H. da Jornada, Diana Y. Qiu, and Steven G. Louie, "Nonuniform sampling schemes of the brillouin zone for many-electron perturbation-theory calculations in reduced dimensionality," Phys. Rev. B **95**, 035109 (2017).

[13] Bradford A. Barker, Jack Deslippe, Johannes Lischner, Manish Jain, Oleg V. Yazyev, David A. Strubbe, and Steven G. Louie, "Spinor gw/bethe-salpeter calculations in berkeleygw: Implementation, symmetries, benchmarking, and performance," Phys. Rev. B **106**, 115127 (2022).

[14] Michael Rohlfing and Steven G. Louie, "Electron-hole excitations and optical spectra from first principles," Phys. Rev. B **62**, 4927–4944 (2000).

[15] Lun Yue, Richard Hollinger, Can B. Uzundal, Bailey Nebgen, Ziyang Gan, Emad Najafidehaghani, Antony George, Christian Spielmann, Daniil Kartashov, Andrey Turchanin, Diana Y. Qiu, Mette B. Gaarde, and Michael Zuerch, "Signatures of multiband effects in high-harmonic generation in monolayer mos_2," Phys. Rev. Lett. **129**, 147401 (2022).

[16] Giovanni Pizzi, Valerio Vitale, Ryotaro Arita, Stefan Blugel, Frank Freimuth, Guillaume Géranton, Marco Gibertini, Dominik Gresch, Charles Johnson, Takashi Koretsune, Julen Ibañez-Azpiroz, Hyungjun Lee, Jae-Mo Lihm, Daniel Marchand, Antimo Marrazzo, Yuriy Mokrousov, Jamal I Mustafa, Yoshiro Nohara, Yusuke Nomura, Lorenzo Paulatto, Samuel Poncé, Thomas Ponweiser, Junfeng Qiao, Florian Thole, Stepan S Tsirkin, Małgorzata Wierzbowska, Nicola Marzari, David Vanderbilt, Ivo Souza, Arash A Mostofi, and Jonathan R Yates, "Wannier90 as a community code: new features and applications," Journal of Physics: Condensed Matter **32**, 165902 (2020).

[17] F. Cadiz, E. Courtade, C. Robert, G. Wang, Y. Shen, H. Cai, T. Taniguchi, K. Watanabe, H. Carrere, D. Lagarde, M. Manca, T. Amand, P. Renucci, S. Tongay, X. Marie, and B. Urbaszek, "Excitonic linewidth approaching the homogeneous limit in mos_2-based van der waals heterostructures," Phys. Rev. X **7**, 021026 (2017).

[18] C. Attaccalite and M. Gruning, "Nonlinear optics from an ab initio approach by means of the dynamical berry phase: Application to second- and third-harmonic generation in semiconductors," Phys. Rev. B **88**, 235113 (2013).

[19] Jiayuan Cao, Fangshu Li, Ya Bai, Peng Liu, and Ruxin Li, "Inter-half-cycle spectral interference in high-order harmonic generation from monolayer mos2," Opt. Express **29**, 4830–4841 (2021).

[20] A Taghizadeh and T G Pedersen, "Nonlinear excitonic spin hall effect in monolayer transition metal dichalcogenides," 2D Materials **7**, 015003 (2019).

[21] Christian Heide, Yuki Kobayashi, Amalya Johnson, Fang Liu, Tony F. Heinz, David A. Reis, and Shambhu Ghimire, "Probing electron-hole coherence in strongly-driven solids," (2021), arXiv:2109.04508 [physics.optics].

[22] Stepan S. Tsirkin, "High performance wannier interpolation of berry curvature and related quantities with WannierBerri code," npj Computational Materials **7** (2021), 10.1038/s41524-021-00498-5.
